# Supplementary material for: Identifying opioid agonist treatment prescriber networks from health administrative data: A validation study
Source: PLoS One. 2025 May 16;20(5):e0322064. doi: 10.1371/journal.pone.0322064 (PMC12083784; doi:10.1371/journal.pone.0322064)
Supplement: S1 Table — (DOCX) [file pone.0322064.s001.docx]

**S1 Table.** Characteristics of the 350 different networks created each calendar year.

| Year | **2013** | **2014** | **2015** | **2016** |
| --- | --- | --- | --- | --- |
| Number of vertices | 102 | 99 | 107 | 115 |
| Lone vertices | 1 (0 - 32) | 1 (1 - 22) | 2 (1 - 17) | 1 (1 - 24) |
| Number of edges | 903 (431 - 1417) | 1049 (456 - 1648) | 1169 (485 - 1829) | 1302 (625 - 2037) |
| Transitivity | 0.55 (0.44 - 0.64) | 0.59 (0.47 - 0.68) | 0.6 (0.47 - 0.67) | 0.59 (0.54 - 0.67) |
| Max degree | 69 (51 - 78) | 77 (56 - 89) | 79 (62 - 90) | 82 (57 - 93) |
| Average degree | 17.7 (8.5 - 27.8) | 21.2 (9.2 - 33.3) | 21.9 (9.1 - 34.2) | 22.6 (10.9 - 35.4) |
| Number of communities detected | 39 (18 - 60) | 34 (18 - 55) | 37.5 (19 - 58) | 37 (19 - 62) |
| Lone communities | 31 (4 - 47) | 22 (5 - 44) | 19.5 (4 - 40) | 24 (6 - 46) |
| Year | **2017** | **2018** | **2019** | **2020** |
| Number of vertices | 114 | 114 | 115 | 114 |
| Lone vertices | 2 (1 - 20) | 2 (1 - 20) | 1 (1 - 27) | 0 (0 - 25) |
| Number of edges | 1307 (601 - 2092) | 1354 (590 - 2108) | 1306 (575 - 2020) | 1146 (501 - 1824) |
| Transitivity | 0.6 (0.48 - 0.67) | 0.59 (0.47 - 0.67) | 0.57 (0.46 - 0.68) | 0.56 (0.46 - 0.66) |
| Max degree | 83 (57 - 96) | 87 (60 - 95) | 79 (57 - 89) | 68 (47 - 82) |
| Average degree | 22.9 (10.5 - 36.7) | 23.7 (10.4 - 37) | 22.7 (10 - 35.1) | 20.1 (8.8 - 32) |
| Number of communities detected | 38 (18 - 61) | 34 (16 - 58) | 34 (17 - 55) | 36 (13 - 59) |
| Lone communities | 20 (6 - 41) | 20 (5 - 40) | 24.5 (4 - 39) | 25 (3 - 40) |
