# Supplementary material for: Identifying opioid agonist treatment prescriber networks from health administrative data: A validation study
Source: PLoS One. 2025 May 16;20(5):e0322064. doi: 10.1371/journal.pone.0322064 (PMC12083784; doi:10.1371/journal.pone.0322064)
Supplement: S2 Table — (DOCX) [file pone.0322064.s002.docx]

**S2 Table.** Summary of characteristics for network-identified clinics and administrative identified clinics between 2013 and 2019.

|  | Network identified communities | Administrative identified clinics |
| --- | --- | --- |
| **2013 clinics** | (n = 31) | (n = 52) |
| No. physicians | 3 (1 - 6) | 3 (2 - 4) |
| No. clients | 126 (4 - 550) | 126 (12 - 261) |
| Physicians’ client load | 26 (2 - 154) | 51 (11 - 98) |
| No. ties | 18 (6 - 40) | 12 (7 - 18) |
| Recall (% of physicians in the network-identified clinic that are in the true clinic) | 0.6 (0.23 - 0.92) | ---------- |
| 1-purity (% of physician in the network-identified clinic that are not in the true clinic) | 0 (0 - 0.31) | ---------- |
| F-measure (harmonic mean of purity and recall values; maximum value of 1 indicates perfect agreement). | 0.67 (0.33 - 0.85) | ---------- |
| **2014 clinics** | (n = 31) | (n = 56) |
| No. physicians | 2 (1 - 5) | 2 (1 - 4) |
| No. clients | 234 (9 - 578) | 128 (15 - 267.25) |
| Physicians’ client load | 55 (9 - 214) | 57 (11.125 - 115.5) |
| No. ties | 23 (12 - 46) | 15 (9 - 21.625) |
| Recall (% of physicians in the network-identified clinic that are in the true clinic) | 0.6 (0.23 - 0.79) | ---------- |
| 1-purity (% of physician in the network-identified clinic that are not in the true clinic) | 0 (0 - 0.17) | ---------- |
| F-measure (harmonic mean of purity and recall values; maximum value of 1 indicates perfect agreement). | 0.67 (0.33 - 0.83) | ---------- |
| **2015 clinics** | (n = 38) | (n = 58) |
| No. physicians | 2 (1 - 4) | 3 (1 - 4) |
| No. clients | 181 (18 - 512) | 139 (74 - 275.25) |
| Physicians’ client load | 78 (20 - 310) | 53.25 (21 - 100.75) |
| No. ties | 30 (13 - 54) | 16.25 (10 - 24.875) |
| Recall (% of physicians in the network-identified clinic that are in the true clinic) | 0.5 (0.25 - 0.75) | ---------- |
| 1-purity (% of physician in the network-identified clinic that are not in the true clinic) | 0 (0 - 0.11) | ---------- |
| F-measure (harmonic mean of purity and recall values; maximum value of 1 indicates perfect agreement). | 0.57 (0.37 - 0.79) | ---------- |
| **2016 clinics** | (n = 38) | (n = 60) |
| No. physicians | 2 (1 - 4.75) | 2.5 (1 - 4) |
| No. clients | 149 (15 - 598) | 148.5 (31.25 - 298) |
| Physicians’ client load | 74 (10 - 292) | 73 (13.875 - 145.25) |
| No. ties | 31 (10 - 55) | 16.5 (5 - 24.125) |
| Recall (% of physicians in the network-identified clinic that are in the true clinic) | 0.5 (0.26 - 0.79) | ---------- |
| 1-purity (% of physician in the network-identified clinic that are not in the true clinic) | 0 (0 - 0.2) | ---------- |
| F-measure (harmonic mean of purity and recall values; maximum value of 1 indicates perfect agreement). | 0.63 (0.4 - 0.79) | ---------- |
| **2017 clinics** | (n = 38) | (n = 60) |
| No. physicians | 2 (1 - 3.75) | 2 (1 - 4) |
| No. clients | 148 (14 - 551) | 173 (74 - 317.75) |
| Physicians’ client load | 106 (9 - 338) | 83.5 (27.125 - 172) |
| No. ties | 31 (9 - 57) | 17.25 (7.75 - 28.125) |
| Recall (% of physicians in the network-identified clinic that are in the true clinic) | 0.46 (0.2 - 0.95) | ---------- |
| 1-purity (% of physician in the network-identified clinic that are not in the true clinic) | 0 (0 - 0) | ---------- |
| F-measure (harmonic mean of purity and recall values; maximum value of 1 indicates perfect agreement). | 0.57 (0.33 - 0.75) | ---------- |
| **2018 clinics** | (n = 33) | (n = 59) |
| No. physicians | 4 (1 - 5) | 2 (1 - 4) |
| No. clients | 320 (8 - 627) | 172 (82.5 - 318) |
| Physicians’ client load | 155 (8 - 358) | 91.5 (21.5 - 156.5) |
| No. ties | 34 (11 - 54) | 16.5 (8.75 - 26) |
| Recall (% of physicians in the network-identified clinic that are in the true clinic) | 0.5 (0.4 - 1) | ---------- |
| 1-purity (% of physician in the network-identified clinic that are not in the true clinic) | 0 (0 - 0.38) | ---------- |
| F-measure (harmonic mean of purity and recall values; maximum value of 1 indicates perfect agreement). | 0.67 (0.46 - 0.73) | ---------- |
| **2019 clinics** | (n = 31) | (n = 60) |
| No. physicians | 3 (1 - 5.5) | 2 (1 - 4) |
| No. clients | 330 (7 - 690) | 182 (92 - 317.25) |
| Physicians’ client load | 134 (6 - 280) | 90 (37.25 - 134.25) |
| No. ties | 26 (9 - 51) | 16.25 (10 - 26) |
| Recall (% of physicians in the network-identified clinic that are in the true clinic) | 0.6 (0.31 - 0.93) | ---------- |
| 1-purity (% of physician in the network-identified clinic that are not in the true clinic) | 0 (0 - 0.33) | ---------- |
| F-measure (harmonic mean of purity and recall values; maximum value of 1 indicates perfect agreement). | 0.67 (0.4 - 0.76) | ---------- |
