# Supplementary material for: Identifying opioid agonist treatment prescriber networks from health administrative data: A validation study
Source: PLoS One. 2025 May 16;20(5):e0322064. doi: 10.1371/journal.pone.0322064 (PMC12083784; doi:10.1371/journal.pone.0322064)
Supplement: S3 Table — (DOCX) [file pone.0322064.s003.docx]

**S3 Table.** Concordance between physician pairs’ assigned network identified community and the gold-standard administrative identified clinic for different algorithm settings.

|  | Concordance statistics | | | | Validity statistics | | | |
| --- | --- | --- | --- | --- | --- | --- | --- | --- |
|  | Overall agree | Positive agree | Negative agree | Gwet’s AC_1_  (95% CI) | Sensitivity | Specificity | Positive predictive value | Negative predictive value |
| **Physician-clinic connection definition*** |  |  |  |  |  |  |  |  |
| Any clinic | 0.46 | 0.11 | 0.62 | 0.08 (0.07, 0.09) | 0.06 (0.06, 0.06) | 1.00 (1.00, 1.00) | 0.89 (0.88, 0.91) | 0.45 (0.44, 0.45) |
| Clinic with majority of clients | 0.97 | 0.41 | 0.98 | 0.96 (0.96, 0.96) | 0.54 (0.51, 0.57) | 0.98 (0.97, 0.98) | 0.34 (0.31, 0.36) | 0.99 (0.99, 0.99) |
| 15% or more clients attached to clinic | 0.96 | 0.49 | 0.98 | 0.95 (0.95, 0.96) | 0.45 (0.43, 0.47) | 0.98 (0.98, 0.98) | 0.54 (0.52, 0.57) | 0.98 (0.97, 0.98) |
| 30% or more | 0.96 | 0.44 | 0.98 | 0.96 (0.96 ,0.96) | 0.53 (0.50, 0.55) | 0.98 (0.98, 0.98) | 0.38 (0.36, 0.40) | 0.99 (0.99, 0.99) |
| 45% or more | 0.96 | 0.42 | 0.98 | 0.96 (0.96, 0.96) | 0.54 (0.50, 0.56) | 0.98 (0.97, 0.98) | 0.34 (0.32, 0.37) | 0.99 (0.99, 0.99) |
| **Community detection algorithm^†^** |  |  |  |  |  |  |  |  |
| MM with 1 iteration | 0.93 | 0.44 | 0.96 | 0.92 (0.92, 0.92) | 0.62 (0.60, 0.94) | 0.94 (0.94, 0.95) | 0.34 (0.32, 0.35) | 0.98 (0.98, 0.98) |
| MM with 2 iterations | 0.96 | 0.49 | 0.98 | 0.96 (0.95, 0.96) | 0.45 (0.43, 0.47) | 0.98 (0.98, 0.98) | 0.54 (0.52, 0.57) | 0.98 (0.97, 0.98) |
| MM with 3 iterations | 0.96 | 0.41 | 0.98 | 0.96 (0.96, 0.96) | 0.30 (0.29, 0.32) | 0.99 (0.99, 0.99) | 0.63 (0.60, 0.66) | 0.97 (0.97, 0.97) |
| MM with 4 iterations | 0.96 | 0.29 | 0.98 | 0.96 (0.96, 0.96) | 0.18 (0.17, 0.20) | 1.00 (1.00, 1.00) | 0.66 (0.63, 0.70) | 0.96 (0.96, 0.97) |
| Louvain | 0.75 | 0.23 | 0.85 | 0.66 (0.66, 0.67) | 0.82 (0.80, 0.83) | 0.75 (0.75, 0.75) | 0.13 (0.13, 0.14) | 0.99 (0.99, 0.99) |
| Walktrap | 0.73 | 0.19 | 0.84 | 0.63 (0.62, 0.63) | 0.73 (0.72, 0.75) | 0.73 (0.73, 0.73) | 0.11 (0.11, 0.12) | 0.98 (0.98, 0.99) |
| Label Propagation | 0.52 | 0.14 | 0.66 | 0.19 (0.18, 0.19) | 0.92 (0.90, 0.93) | 0.50 (0.49, 0.50) | 0.08 (0.08, 0.08) | 0.99 (0.99, 0.99) |

Abbreviations: MM: Modularity maximization.

*True physician-clinic pairings may have a physician at more than one clinic; however, the network can only group a physician to one community. To test how this impacted results, we had several different definitions to define a “true” physician-clinic connection. The presented results are under modularity maximization algorithm with 2 iterations and a relative threshold of top 80% of tie weights required to define a connection in the network.

^†^Clinics in the network were assigned through community detection algorithms. We included multiple algorithms to test how this impacted results. The presented results are with ‘true’ physician-clinic connections defined as those with at least 15% or more of a physicians’ total patients and a relative threshold of top 80% of tie weights required to define a connection in the network.
