# Supplementary figures and images for: Identifying opioid agonist treatment prescriber networks from health administrative data: A validation study
Source: PLoS One. 2025 May 16;20(5):e0322064. doi: 10.1371/journal.pone.0322064 (PMC12083784; doi:10.1371/journal.pone.0322064)

**S1 Fig.** Sensitivity and positive predictive value estimates

**
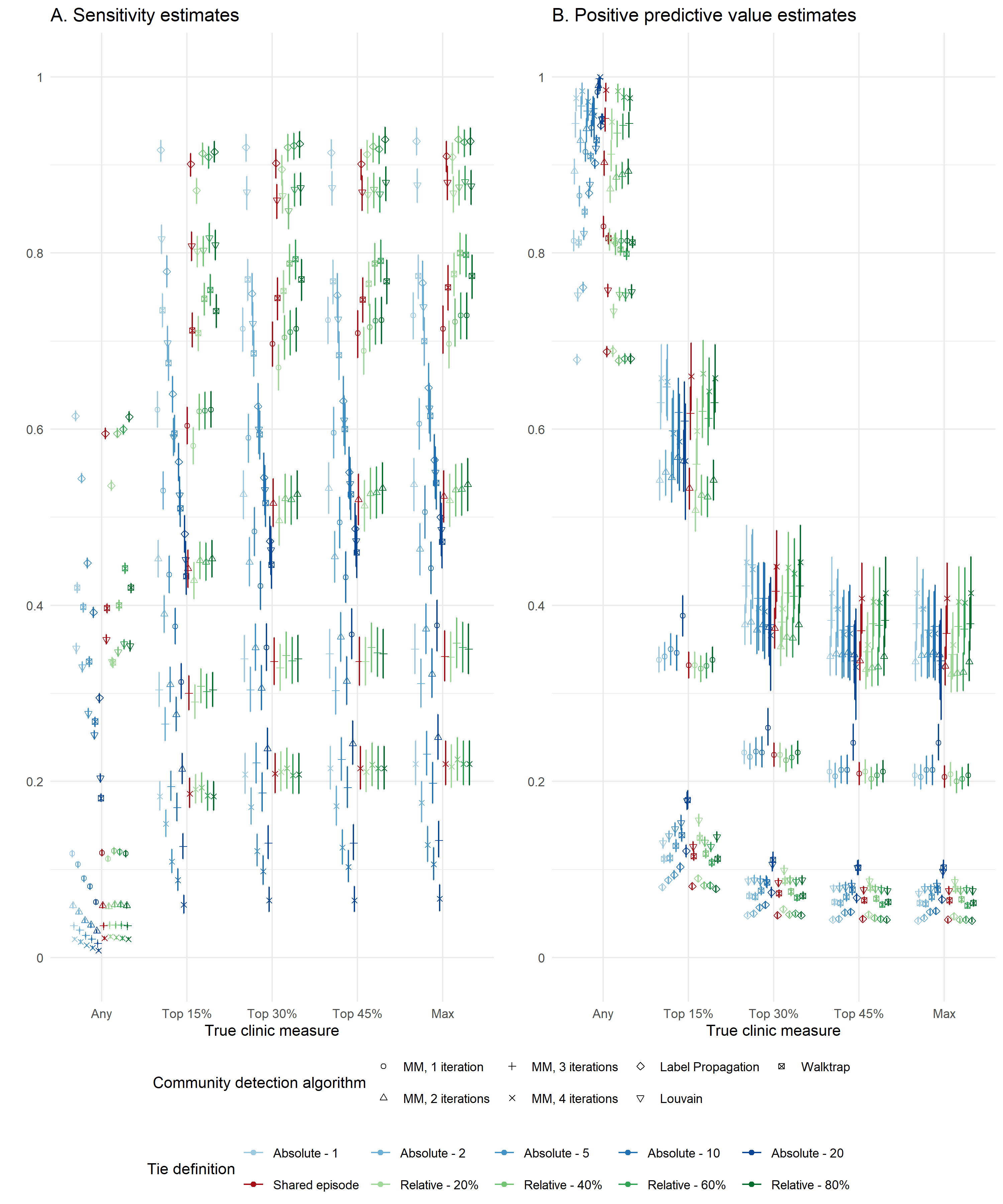
**

Supplement: S1 Fig — (DOCX) [file pone.0322064.s004.docx]

**S2 Fig.** Specificity and negative predictive value estimates

**
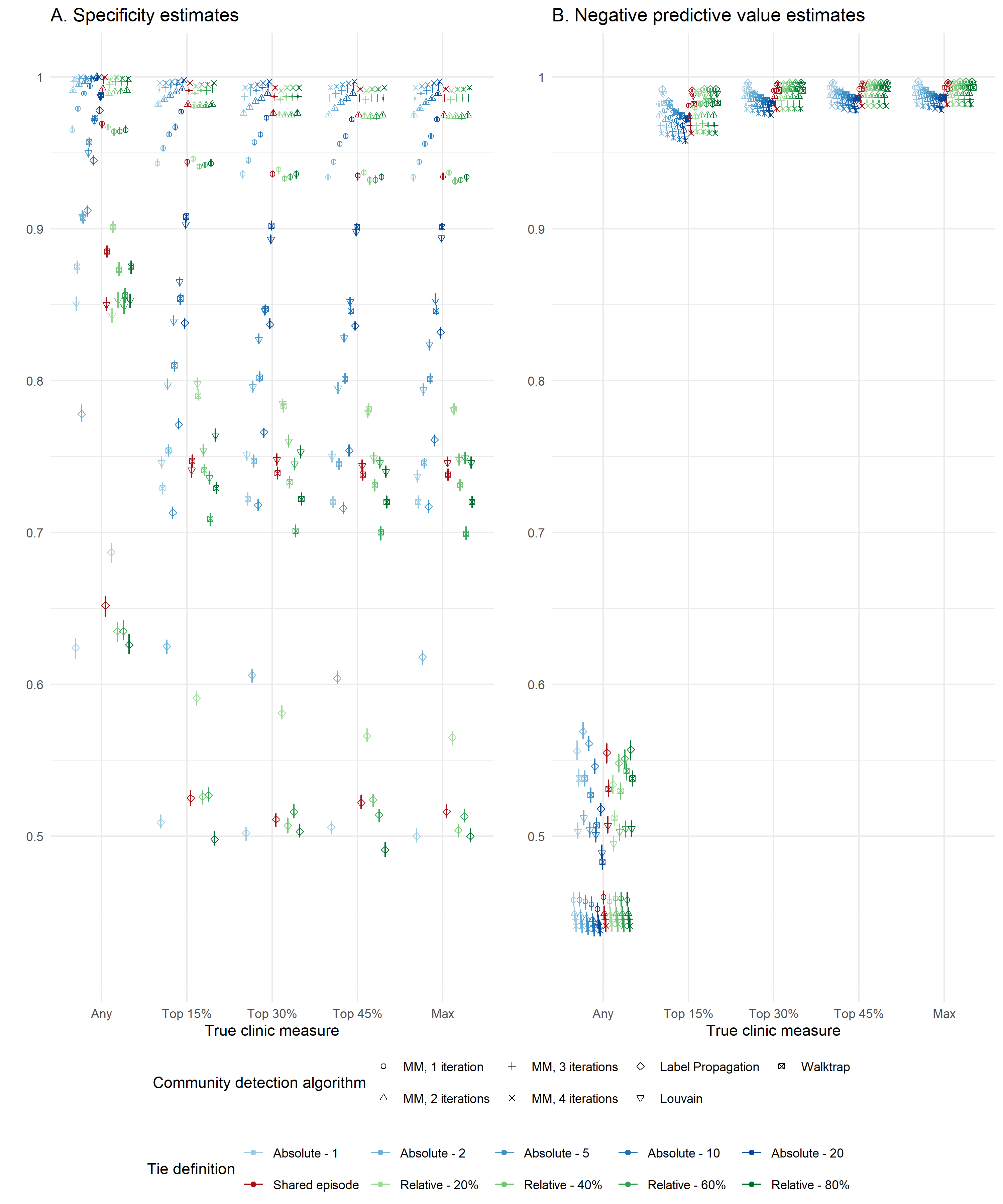
**

Supplement: S2 Fig — (DOCX) [file pone.0322064.s005.docx]
